# *In situ* growth of N-doped carbon nanotubes from the products of graphitic carbon nitride etching by nickel nanoparticles†

**DOI:** 10.1039/d3na00983a

**Published:** 2024-02-21

**Authors:** Mariusz Pietrowski, Emilia Alwin, Michał Zieliński, Sabine Szunerits, Agata Suchora, Robert Wojcieszak

**Affiliations:** a Faculty of Chemistry, Adam Mickiewicz University, Poznań Uniwersytetu Poznańskiego 8 61-614 Poznań Poland; b Univ. Lille, CNRS, Centrale Lille Univ. Polytechnique Hauts-de-France, UMR 8520 – IEMN F-59000 Lille France; c Univ. Lille, CNRS, Centrale Lille, Univ. Artois UMR 8181 – UCCS – Unité de Catalyse et Chimie du Solide F-59000 Lille France robert.wojcieszak@univ-lille.fr; d Université de Lille and CNRS, L2CM UMR 7053 Nancy F54000 France

## Abstract

The *in situ* growth of N-doped multi-walled carbon nanotubes (N-MWCNTs) from the products of graphitic carbon nitride (g-C_3_N_4_) etching by Ni nanoparticles in a hydrogen atmosphere has been confirmed for the first time. During the etching process of g-C_3_N_4_, the building blocks, notably methane, ammonia, and hydrogen cyanide, are formed. The formation of N-MWCNTs was confirmed by Raman spectroscopy, X-ray photoelectron spectroscopy (XPS), X-ray diffraction (XRD) and scanning (SEM) and transmission electron microscopy (TEM). A sponge-like carbonaceous structure was obtained with a specific surface area of 384 m^2^ g^−1^ from initial g-C_3_N_4_ (32 m^2^ g^−1^).

## Abstract 2

The products of g-C_3_N_4_ etching are CH_4_, NH_3_, and HCN, which are used as building materials for N-doped carbon nanotubes without any external source of carbon or nitrogen.

## Graphical abstract

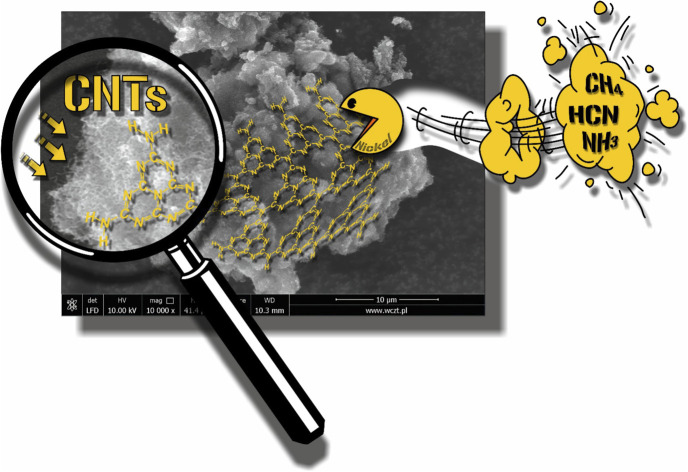

## 1 Introduction

Graphitic carbon nitride (g-C_3_N_4_) has gained widespread recognition as a promising two-dimensional metal-free photocatalyst, primarily due to its impressive optical properties, environmentally friendly nature, and exceptional chemical and thermal stability.^1,2^ Nevertheless, its practical application in photocatalytic hydrogen evolution has been hindered by inherent limitations. Its narrow light absorption range, rapid recombination of photoinduced electron–hole pairs, and its relatively low specific surface area have collectively contributed to suboptimal hydrogen evolution activity.^3,4^ g-C_3_N_4_ is a layered polymer analogous to graphite.^5,6^ In contrast to the single-layer structure of graphene,^7,8^ graphite is a bulk material with multiple layers of graphene stacked together. The layers are formed of joined units of heptazine. As a typical semiconductor with a smaller energy gap than popular photocatalysts, g-C_3_N_4_ can be used in the photodegradation of organic pollutants or photoreduction of water to hydrogen under visible light irradiation. In addition to its several advantages, g-C_3_N_4_ has a low specific surface area (SSA) of 10–30 m^2^ g^−1^.^9^ Researchers worldwide have been actively exploring innovative strategies to overcome these challenges and unlock the full potential of g-C_3_N_4_. To address the issue of limited specific surface area, one fundamental approach has been to engineer g-C_3_N_4_ with distinct morphologies, such as nanotubes^10^ and hollow nanospheres.^11,12^ Additionally, given the layered structure of g-C_3_N_4_, scientists have delved into methods like exfoliation using liquid phases or steam reforming as effective means of obtaining few-layer g-C_3_N_4_.^13,14^ While these strategies have shown promise, the process of employing templates to regulate g-C_3_N_4_ morphologies and the exfoliation techniques themselves can be intricate and time-consuming. Furthermore, many exfoliated g-C_3_N_4_ nanosheets tend to exhibit small lateral dimensions, posing challenges related to catalyst recovery and limiting their utility in diverse applications, including nanocomposites for reinforcement and gas barrier materials.^6,15^ In the 1970s, Tomita and Tamai^16^ discovered that in the presence of hydrogen, metal nanoparticles etch open trenches in the structure of graphite. Since then, the etching/nanocutting of carbon materials has been observed for Ni, Co, Fe, and Ag (nano)particles several times.^17–20^ Lukas *et al.*^19^ reported that Ni nanoparticles etch open trenches on the surface of carbonaceous materials and create a network of tunnels in their 3D structure resulting in porous graphite. However, this phenomenon of nanocutting has been previously observed only for graphite/graphene,^17–20^ as well as for hexagonal boron nitride,^21^ but never for g-C_3_N_4_.

In this article, we delve into an innovative approach that capitalizes on the products of graphitic carbon nitride (g-C_3_N_4_) etching by nickel nanoparticles, leading to the *in situ* growth of N-doped carbon nanotubes. By exploring this novel method, we aim to provide insights into how g-C_3_N_4_ limitations can be effectively addressed, ultimately leading to the synthesis of g-C_3_N_4_ with a larger specific surface area and higher quality. This approach not only enhances the photocatalytic potential of g-C_3_N_4_ but also opens up new avenues for its application in various fields, further underscoring its versatility and significance in materials science and catalysis. By focusing on this innovative methodology, we hope to contribute to the ongoing efforts to optimize g-C_3_N_4_ for enhanced hydrogen evolution and highlight its potential as a key player in the future of sustainable and efficient photocatalysis.

## 2 Experimental

### 2.1. Materials and methods

#### 2.1.1. Synthesis of g-C_3_N_4_ and the Ni/g-C_3_N_4_ catalyst

Graphitic carbon nitride (g-C_3_N_4_) was obtained by pyrolysis of dicyandiamide (DCDA) (Sigma-Aldrich, 99%). The scheme of the synthesis method is presented in Fig. S1.† A portion of 4 g DCDA was placed in a quartz crucible of 50 mL in capacity, covered with a lid and heated in a muffle furnace for 4 h at 600 °C (heating rate of 10 °C min^−1^). The crucible was left in the furnace to cool down to room temperature. The obtained g-C_3_N_4_ of canary-yellow colour was ground in an agate mortar to fine powder. On the surface of the so-prepared g-C_3_N_4_, from a water solution, Ni(NO_3_)_2_·6H_2_O was introduced by wet-impregnation. The amount of nickel nitrate was adjusted so that to obtain the contents of 5.0, 1.0, 0.5 and 0.25 wt% Ni on g-C_3_N_4_. The suspension of g-C_3_N_4_ in a water solution of nickel nitrate of an appropriate concentration was stirred on a magnetic stirrer for 2 h. Then water was evaporated on a rotary evaporator. The obtained samples were dried overnight at 80 °C. The samples of the catalysts and g-C_3_N_4_ were maintained in an exicator over 4 Å molecular sieves. Apart from the above sample, another one was made with nickel nitrate introduced on the surface of g-C_3_N_4_ also by incipient-wetness and yet another sample was obtained as a physical mixture of nickel nitrate and g-C_3_N_4_. In these two samples the loading with nickel was 1 wt%.

#### 2.1.2. Reduction of g-C_3_N_4_ and the Ni/g-C_3_N_4_ catalyst

The sample of g-C_3_N_4_ and nickel catalysts were reduced in a tube furnace, in hydrogen flow at 50 mL min^−1^ and at a heating rate of 10 °C min^−1^. The reduced g-C_3_N_4_ was labelled as g-C_3_N_4_-r, while the catalyst as Ni/g-C_3_N_4_. The yield of the product obtained after the reduction was calculated from the formula *Y* [%] = (*m*_r_/*m*_i_) × 100%, where *m*_r_ is the mass of the reduced product and *m*_i_ is the initial mass of the sample.

### 2.2. Characterization of the samples

#### 2.2.1. Specific surface area and porosity measurements

The specific surface area (SSA) was determined by the Brunauer–Emmett–Teller (BET) method using a Micromeritics ASAP 2010 surface area and porosity analyser (surface areas were obtained from N_2_ adsorption isotherms collected at 77 K). Isotherms are shown in Fig. S2.†

#### 2.2.2. Scanning electron microscopy (SEM) and transmission electron microscopy (TEM) imaging

An FEI Helios NanoLab 660 (Thermo Fisher Scientific, Waltham, MA, USA) electron microscope was used to obtain SEM images. A Hitachi HT7700 microscope (Hitachi, Tokyo, Japan) at an accelerating voltage of 100 kV was used to record TEM images.

#### 2.2.3. X-ray powder diffraction (XRD)

The powder X-ray diffraction patterns of the samples were obtained using a Bruker D8 Advance diffractometer by using CuKα radiation. The XRD data were collected over a 2-theta range of 10–35° with a step size of 0.01° and a scanning time rate of 5 seconds.

#### 2.2.4. X-ray photoelectron spectroscopy (XPS)

XPS measurements were made with a Kratos Axis Ultra spectrometer (Kratos Analytical, Manchester, UK). The excitation source was a monochromatized aluminium X-ray source (Al Kα (1486.6 eV) operated at 10 mA and 15 kV). The charge referencing method used was the C (C, H) component of the C 1s peak of adventitious carbon fixed at 284.5 eV. Spectroscopic data were processed by the CasaXPS ver. 2.3.17PR1.1 software (Casa Software Ltd, UK), using a peak-fitting routine with Shirley background and asymmetrical Voigt functions.

#### 2.2.5. Raman spectroscopy

The Raman spectra were recorded on a XploraRaman confocal microscope from HORIBA Jobin Yvon. A 785 nm diode laser was used to excite the samples through a macro device connected to a multipass cell holder.

## 3 Results and discussion

**Fig. 1:**
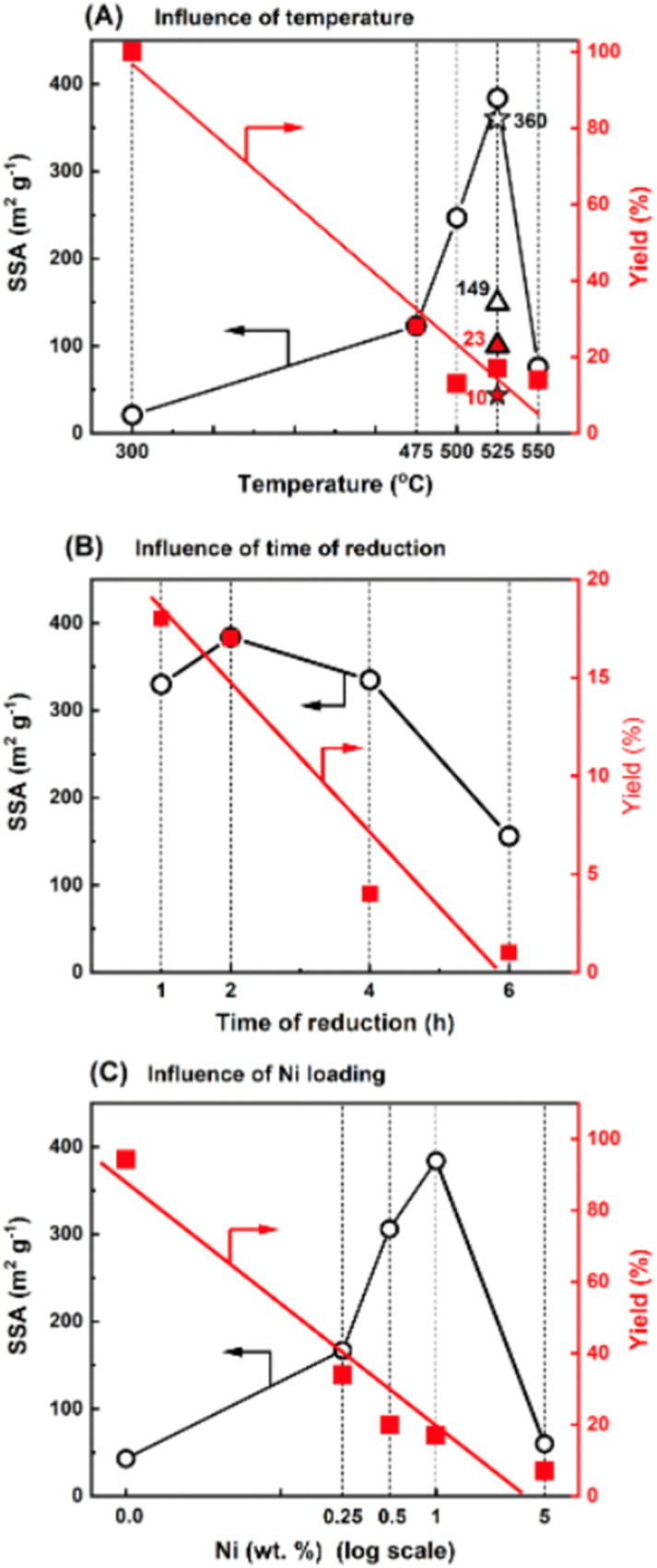
Influence of temperature, time and metal loading during reduction on the specific surface area of Ni/g-C_3_N_4_: effects of reduction temperature (A), reduction time at 525 °C (B), and metal loading (C) on SSA and yield of Ni/g-C_3_N_4_-r samples prepared by the wet-impregnation method. In (A), the asterisk and triangle represent catalysts prepared by the incipient wetness method (IW) and by physically mixing (PM) of nickel nitride and g-C_3_N_4_, respectively.

In this study, we used Ni nanoparticles for g-C_3_N_4_ structure nanoengineering through etching/nanocutting to enlarge its specific surface area (SSA) by creating a porous structure. Using the wet-impregnation method (wet-I), nickel(ii) nitrate was introduced onto the surface of g-C_3_N_4_. Reduction of this material under hydrogen conditions at 475 °C significantly increased the SSA from 32 m^2^ g^−1^ (for g-C_3_N_4_) to 123 m^2^ g^−1^ in the novel materials. Because the nickel content was only 1 wt%, the high increase in the SSA is notable due to the increase in the SSA of g-C_3_N_4_ and the formation of a novel structure. The effects of temperature, time of reduction, and metal loading were evaluated. Fig. 1A illustrates the effect of reduction temperature on the SSA of the sample with 1 wt% Ni deposited on g-C_3_N_4_ (Ni/g-C_3_N_4_-r) after 2 h. With increasing reduction temperature, the SSA value increased and reached a maximum of 384 m^2^ g^−1^ at 525 °C. An increase in the reduction temperature to 550 °C decreased the SSA of the catalyst. This decrease may be due to deeper destruction of the g-C_3_N_4_ structure that occurs at high temperatures. The impact of the reduction time on the SSA was evaluated for 1 wt% Ni/g-C_3_N_4_ at 525 °C and is illustrated in Fig. 1B. The largest SSA was obtained after 2 h of reduction. For the optimized reduction temperature and time (525 °C, 2 h), the effect of metal loading was tested for the samples containing 0.25, 0.5, 1.0, and 5.0 wt% Ni (Fig. 1C). The largest SSA was obtained for the sample with 1.0 wt% Ni, although a significant increase in SSA (from 32 to 167 m^2^ g^−1^) was observed already for the lowest metal loading of 0.25 wt%. Apart from the SSA, the yield of the final product was also monitored (the axes on the right-hand side of the plots shown in Fig. 1). The yield of the product decreased with increasing temperature (Fig. 1A), time of reduction (Fig. 1B), and metal loading (Fig. 1C). Under the optimized conditions (525 °C, 2 h, 1.0 wt% Ni) the yield of the final product was 17%. The mass loss was accompanied by intensive emission of the products of g-C_3_N_4_ hydrogenation/hydrogenolysis: methane, ammonia, and hydrogen cyanide (identified by mass spectrometry; Fig. S6 and S7, Table S1†). The effect of the nickel deposition method on g-C_3_N_4_ was also investigated. Two additional methods of Ni deposition were applied: incipient wetness method (IW) and physical mixing (PM) of g-C_3_N_4_ with nickel nitrate obtained by grinding both components in an agate mortar. The results are presented in Fig. 1A, in which the asterisk and triangle denote the catalysts prepared by the IW and PM methods, respectively. The behaviors of the catalysts obtained by IW and wet-I were the same; hence, these two methods did not affect the SSA of g-C_3_N_4_. For the sample obtained by the physical mixing, the metal contact with the g-C_3_N_4_ surface was significantly worse than that of the metal introduced from the solution. Thus, the increase in SSA was lower (149 m^2^ g^−1^) than that of the catalysts obtained by IW and wet-I. Moreover, the product yield was higher (23%) in the PM method than in IW and wet-I methods.

According to the experimental results, the factors sufficient for the initiation of the processes and responsible for the increase in the SSA of g-C_3_N_4_ are the presence of metal and hydrogen at an elevated temperature. In another experiment, the effect of the reduction on pristine g-C_3_N_4_ was studied. The reduction effect was small, but noticeable. The SSA increased from 32 to 43 m^2^ g^−1^, while the final product yield reached 94.2%. Niu *et al.*^22,23^ established that the reduction of g-C_3_N_4_ in hydrogen at 540 °C eliminated amine groups and generated nitrogen vacancies; however, the reduction did not result in significant changes in the crystal structure of g-C_3_N_4_.^22^ Similarly, the exposure of g-C_3_N_4_ to hydrogen at 550 °C has been reported to delaminate carbon nitrate.^23^

**Fig. 2:**
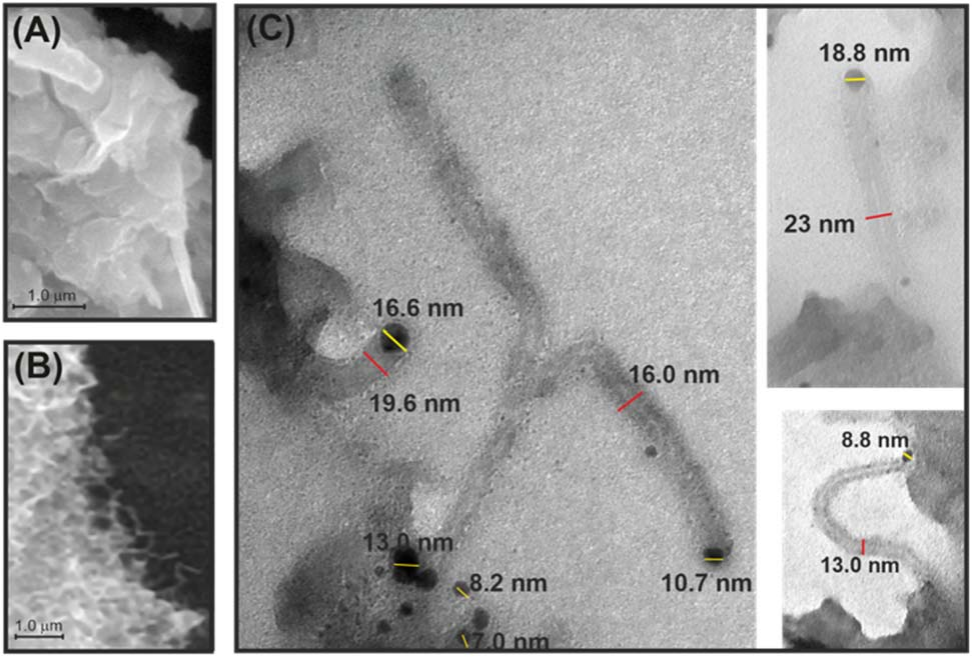
SEM micrographs of reduced g-C_3_N_4_ (denoted as g-C_3_N_4_-r) (A) and the Ni/g-C_3_N_4_-r sample (B), and TEM images of MWCNTs in the Ni/g-C_3_N_4_-r sample (C).

Fig. 2A and B show scanning electron microscopy images of g-C_3_N_4_ and the 1 wt% Ni/g-C_3_N_4_-r sample after reduction in hydrogen at 525 °C for 2 h. No reduction effect on the structure of pristine g-C_3_N_4_ was observed. In contrast, the structure of the Ni/g-C_3_N_4_-r sample after the reduction resembled that of a porous sponge (cotton wad) with clearly marked fibrous structures (Fig. 2B and S1†). The transmission electron microscopy images in Fig. 2C show that these structures are typical of carbon nanotubes (CNTs). The presence of internal channels and nanotube diameters larger than 10 nm indicates that they are the multi-walled carbon nanotubes (MWCNTs).^24^ The nanotubes are terminated with a metal crystallite, implying that their growth mechanism must have been the tip growth mode.^24^ The diameters of the MWCNTs varied from 9 to 36 nm, and their length reached 300 nm. The average size of nickel nanoparticles is 7.5 ± 3.8 nm and is slightly larger than in the sample reduced at 300 °C (TEM images and particle size histograms – Fig. S2†). The growth of CNTs in the system studied is highly probable because Ni nanoparticles (in addition to iron and cobalt) are used for the synthesis of MWCNTs through catalytic chemical vapor deposition (CCVD).^25^ Moreover, the most frequently used source of carbon, methane, was observed in the products of g-C_3_N_4_ etching (Fig. S4†). The presence of MWCNTs was also recorded for the Co/g-C_3_N_4_-r sample, although at a lower amount (not presented).

**Fig. 3:**
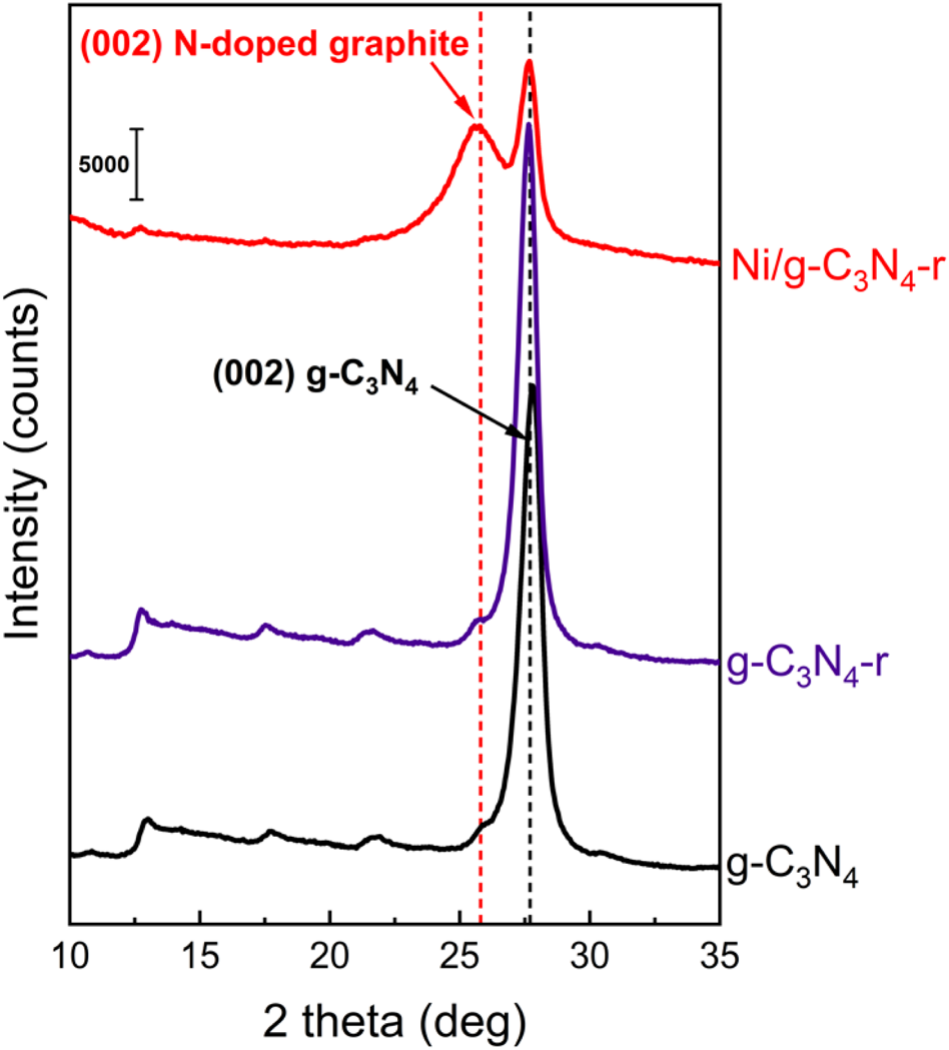
X-ray powder diffraction patterns of pristine g-C_3_N_4_, reduced g-C_3_N_4_-r and the reduced Ni/g-C_3_N_4_-r sample.

The X-ray diffraction (XRD) diffractogram of pristine g-C_3_N_4_, which is a layered material similar to graphite, shows a characteristic reflection at 2*θ* of 27.6°, corresponding to an interlayer spacing of ∼3.2 Å (Fig. 3). As expected, the XRD pattern was practically unchanged after the reduction of pristine g-C_3_N_4_ in hydrogen, which is in agreement with the observations of Niu *et al.*^22^ In the XRD diffractogram of the Ni/g-C_3_N_4_-r sample, the intensity of the reflection at 2*θ* of 27.6° significantly decreased, indicating partial destruction of the g-C_3_N_4_ structure from reduction.

Another feature of this diffractogram is the appearance of a new reflection at 2*θ* of 25.7°. This signal originates from the diffraction at the (002) plane of graphitic carbon in MWCNTs and corresponds to an interlayer spacing of ∼3.46 Å. However, the spacing does not correspond to purely carbon MWCNTs, because they are characterized by a smaller *d*-spacing of ∼3.36 Å (26.56° 2*θ*).^26^ An increase in the interplanar distance is generally due to the CNT admixture with foreign atoms.^27^ In this system, we considered the admixture of MWCNTs with nitrogen atoms.

**Fig. 4:**
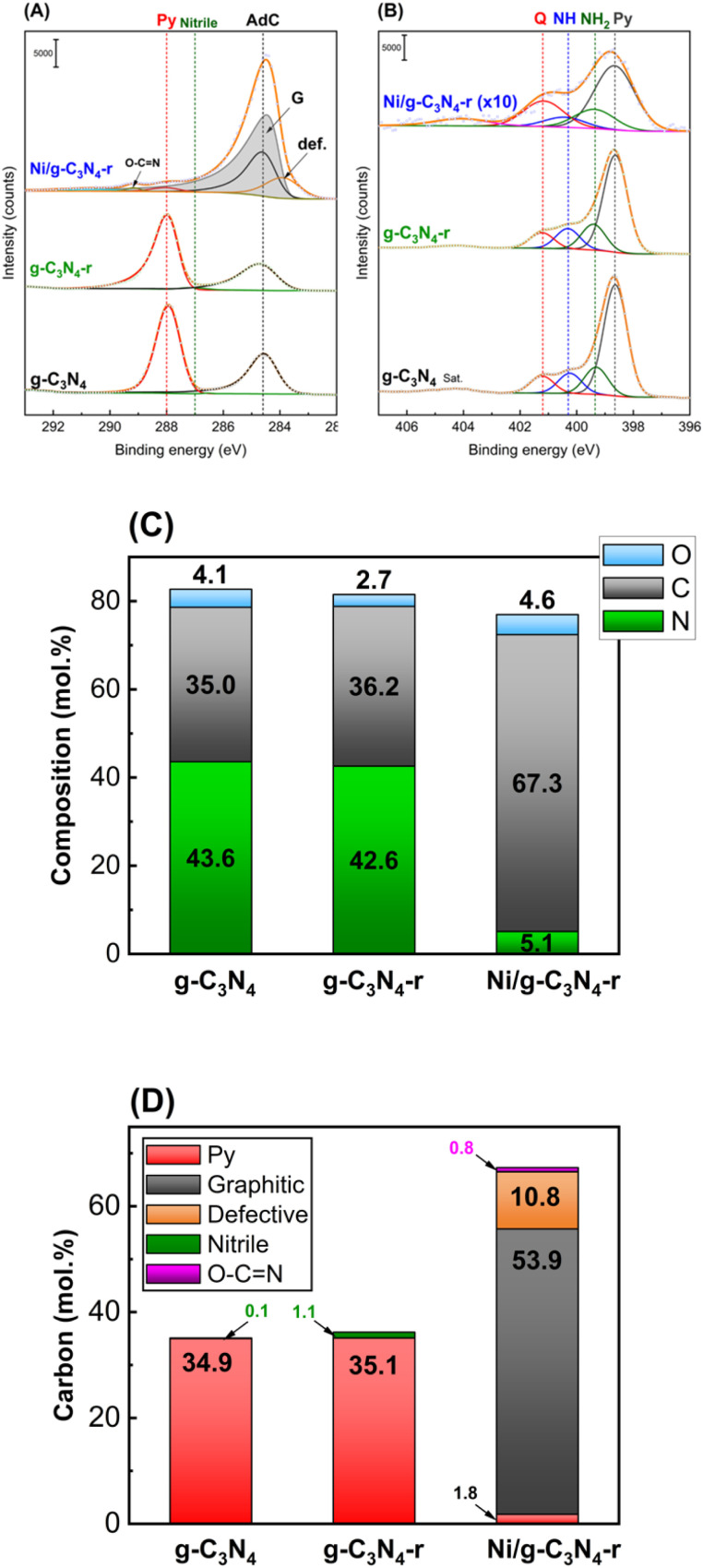
C 1s (A) and N 1s (B) core-level XPS spectra of pristine carbon nitride (g-C_3_N_4_), reduced carbon nitride (g-C_3_N_4_-r), and the Ni/g-C_3_N_4_-r sample reduced in hydrogen at 525 °C for 2 h. Peak labelled as Sat. at ∼404 eV is attributed to the shake-up satellite. In (A), Py, Nitrile, AdC, G, and def. represent pyridinic carbon, nitrile carbon, adventitious carbon, graphitic carbon, and defective carbon. In (B), Q, NH, NH_2_, and Py represent quaternary (graphitic) nitrogen, nitrogen in secondary amine, nitrogen in primary amine, and pyridinic nitrogen in the heptazine ring. (C) and (D) show the results of the quantitative analysis of the XPS spectra; (C) – percentage contribution of C, N, and O (without of AdC); (D) – percentage contribution of particular carbon species determined from the detailed C 1s spectra.

Fig. 4A and B present the C 1s and N 1s core-level X-ray photoelectron spectroscopy (XPS) spectra of the g-C_3_N_4_, g-C_3_N_4_-r and Ni/g-C_3_N_4_-r samples. The C 1s XPS spectrum of g-C_3_N_4_ shows three components, at 284.6, 287.0, and 288.0 eV. These are assigned to adventitious carbon (AdC, sp^2^ carbon), nitrile species –C≡N, and pyridinic carbon in the heptazine ring^28–31^ (Fig. 4A). The reduction of pristine g-C_3_N_4_ under a hydrogen atmosphere at 525 °C (g-C_3_N_4_-r) causes insignificant changes in the C 1s and N 1s spectra, that is, a small decrease in band intensity. Drastic changes were observed in the spectra of the Ni/g-C_3_N_4_-r sample: a new asymmetric band appeared at 284.4 eV, which was assigned to graphitic carbon from MWCNTs. Moreover, a band at 283.9 eV was assigned to the so-called defective carbon, which should be considered as point defects in the graphite lattice.^28^

In addition, the band assigned to pyridinic carbon was significantly diminished. Fig. 4B shows the XPS N 1s core-level spectra. The spectrum of g-C_3_N_4_ shows four characteristic bands at 398.6, 399.3, 400.3, and 401.2 eV, which are assigned to pyridinic nitrogen in the heptazine ring (Py), primary amine (NH_2_), secondary amine (–NH–) and quaternary (graphitic) (Q) nitrogen,^30–34^ respectively. The four bands also appeared in the g-C_3_N_4_-r spectrum, and their intensities did not differ significantly from those in the spectrum of g-C_3_N_4_, that is, only a small increase in the contribution of nitrogen species NH_2_ and NH was observed (Table S2†). In contrast, drastic changes were observed in the N 1s spectrum of the Ni/g-C_3_N_4_-r sample. Because of a strong decrease in the nitrogen content, the N 1s spectrum is shown in Fig. 3B at 10× magnification. In addition, the intensity of the band assigned to quaternary (graphitic) nitrogen significantly increased compared to that assigned to pyridinic nitrogen.

Fig. 4C and D show the elemental compositions of g-C_3_N_4_ (before and after reduction) and Ni/g-C_3_N_4_-r. No significant effect of reduction on the contents of C, N, and O in pristine g-C_3_N_4_ was observed. However, for the Ni/g-C_3_N_4_-r, the contents of C and N significantly changed, and the contributions of carbon and nitrogen increased to 67.3 mol% and decreased to 5.1 mol%, respectively (detailed results of XPS analysis are presented in Table S2†). The high increase in the content of carbon in the Ni/g-C_3_N_4_-r sample was mainly due to the appearance of graphitic carbon, which makes 53.9 mol% of the total carbon content (Fig. 4D) and is the building material of MWCNTs. Simultaneously, the contribution of pyridinic carbon, which is characteristic of the g-C_3_N_4_ structure, decreased twentyfold, that is, from approximately 35 to 1.8 mol%. This indicates that the structure of g-C_3_N_4_ has been considerably damaged.

**Fig. 5:**
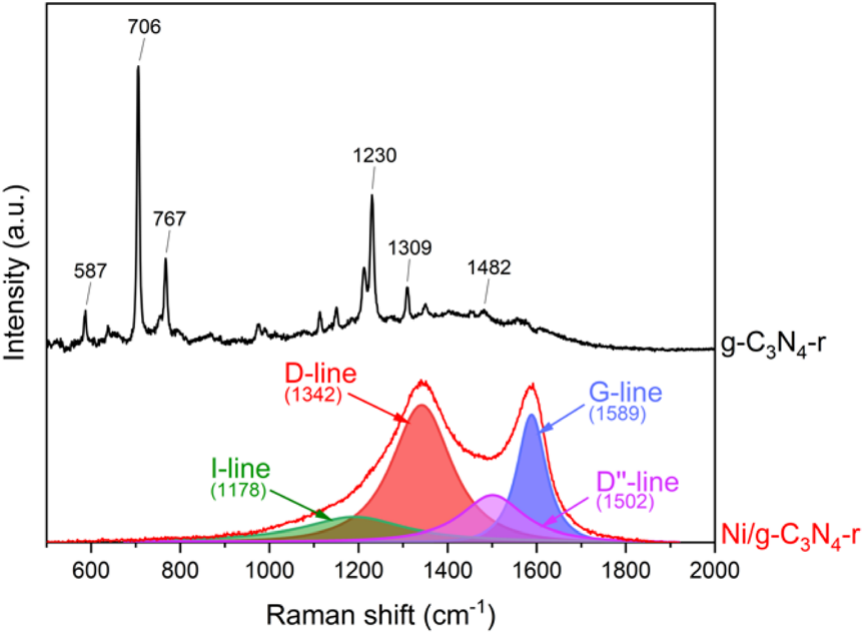
The Raman spectra of g-C_3_N_4_-r and the Ni/g-C_3_N_4_-r sample after H_2_ treatment at 525 °C for 2 h.

Indisputable evidence for the presence of carbon nanotubes in the reduced Ni/g-C_3_N_4_-r material was provided by Raman spectroscopy studies (Fig. 5). In the spectrum of the reduced g-C_3_N_4_-r sample, typical bands of graphitic carbon nitride were observed.^35–37^ The most intense bands of 706 and 1230 cm^−1^ are assigned to the heptazine ring breathing mode and stretching vibration mode of C–N heterocycles, respectively.^38^ In the Ni/g-C_3_N_4_-r spectrum, the characteristic D-band (1342 cm^−1^) and G-band (1589 cm^−1^) were observed, indicating the presence of MWCNTs. In addition, these two bands are broad and overlap to a large extent, indicating a large deformation of the nanotubes. The spectrum of the Ni/g-C_3_N_4_-r sample was deconvoluted, obtaining the best fit using four peaks. In addition to the two main D and G bands, two additional bands were observed at 1178 and 1502 cm^−1^ corresponding to the I-band and D′′-band, respectively.^39^ The I-band is characteristic of N-doped CNTs and the D′′-band is an indicator of the degree of deformation of graphite layers.^40^ Both of these bands are quite intense in our spectrum which indicates a high degree of deformation of MWCNTs. The most commonly used indicator of the degree of defection of CNTs is the ratio of the integrated intensity of the D and G bands (*I*_D_/*I*_G_). For the Ni/g-C_3_N_4_-r sample, it is 2.0 and close to the value obtained for N-doped MWCNTs with ∼5% nitrogen content by Sharifi *et al.*^39^

The synthesis of N-doped MWCNTs obtained from g-C_3_N_4_ was reported by Maślana *et al.*^37^ however, this involved the use of g-C_3_N_4_ as a support for nickel and the use of the traditional CCVD technique and external ethylene as a building block for CNTs. The authors did not succeed in obtaining MWCNTs without external ethylene.

## 4 Conclusions

To summarize, in the presence of hydrogen, Ni nanoparticles were found to induce changes in the g-C_3_N_4_ structure, resulting in a significant increase in the SSA. Our interpretation of the obtained results is that these changes are caused by the etching of g-C_3_N_4_ and involve hydrogenation/hydrogenolysis of the C–N bond in g-C_3_N_4_. Similar to Pac-Man,^41^ the popular computer game from the 1980s, the Ni nanoparticles nibbled graphitic carbon nitride boring tunnels in its structure, which led to the formation of new pores and an increase in the SSA. To date, the phenomenon of channel boring by metal nanoparticles has been observed only for pure carbon materials, mainly graphite and graphene,^17,19,20^ and for hBN,^21^ but never for g-C_3_N_4_. The products of the g-C_3_N_4_ etching are CH_4_, NH_3_, and HCN, which are the building blocks for the MWCNTs grown in the tip growth mode.^24^ Moreover, as observed through the XRD and Raman studies, they are N-doped MWCNTs. The growth of CNTs inside nanocutting channels has been recently reported for highly oriented pyrolytic graphite.^42^ According to our observations, the CNT growth occurs over the entire surface of g-C_3_N_4_ and not only inside the nanocutted tunnels. This leads to the formation of a large-surface-area sponge-like carbon material with a structure like a roll of cotton/pumice. It should be noted that the CNT growth does not require any external source of carbon or nitrogen; the only building blocks are the products of g-C_3_N_4_ etching. The final result is a hybrid material of g-C_3_N_4_ and CNTs, and the procedure can be considered as a new method of synthesizing N-doped MWCNTs or a new class of hybrid carbonaceous materials doped with nitrogen. The unique characteristic of this method is that, in contrast to the commonly used techniques in which nitrogen is introduced into a pure carbon material, here nitrogen is removed from a material rich in nitrogen. The obtained material will have potential application as an electrocatalyst or supercapacitor electrode,^43–45^ catalyst,^46,47^ and catalyst support,^48^ and in the synthesis of new composites.^49^ We hope that the method presented above will inspire other researchers to further modify g-C_3_N_4_; the choice of metal (Fe, Co, Ni), the method of its deposition on the surface of g-C_3_N_4_, and its content in combination with the reaction conditions and nanoparticle size opens wide possibilities for controlling the final structure of N-doped carbon nanomaterials.

## Author contributions

The manuscript was written through contributions of all the authors. All the authors have given approval to the final version of the manuscript. Mariusz Pietrowski: conceptualization, writing – original draft, writing – review and editing, investigation, methodology, visualization. Emilia Alwin: writing – original draft, investigation, methodology. Robert Wojcieszak: writing – review and editing, investigation. Michał Zieliński: writing – review and editing, investigation. Sabine Szunerits: writing – review and editing, investigation. Agata Suchora: investigation.

## Conflicts of interest

There are no conflicts to declare.

## Supplementary Material

NA-006-D3NA00983A-s001
