# Expression of concern: *In situ* growth of N-doped carbon nanotubes from the products of graphitic carbon nitride etching by nickel nanoparticles

**DOI:** 10.1039/d4na90119c

**Published:** 2024-12-05

**Authors:** Mariusz Pietrowski, Emilia Alwin, Michał Zieliński, Sabine Szunerits, Agata Suchora, Robert Wojcieszak

**Affiliations:** a Faculty of Chemistry, Adam Mickiewicz University, Poznań Uniwersytetu Poznańskiego 8 61-614 Poznań Poland; b Univ. Lille, CNRS, Centrale Lille Univ. Polytechnique Hauts-de-France, UMR 8520 – IEMN F-59000 Lille France; c Univ. Lille, CNRS, Centrale Lille, Univ. Artois UMR 8181 – UCCS – Unité de Catalyse et Chimie du Solide F-59000 Lille France robert.wojcieszak@univ-lille.fr; d Université de Lille and CNRS, L2CM UMR 7053 Nancy F54000 France

## Abstract

Expression of concern for ‘*In situ* growth of N-doped carbon nanotubes from the products of graphitic carbon nitride etching by nickel nanoparticles’ by Mariusz Pietrowski *et al.*, *Nanoscale Adv.*, 2024, **6**, 1720–1726, https://doi.org/10.1039/D3NA00983A.

The Royal Society of Chemistry is publishing this expression of concern in order to alert readers that concerns have been raised regarding the reliability of the data. The Royal Society of Chemistry has asked the University of Lille to investigate this matter. An expression of concern will continue to be associated with the article until we receive conclusive evidence regarding the reliability of the reported data.

Dr Jeremy Allen

4th November 2024

Executive Editor, *Nanoscale Advances*

