# Supplementary material for: In situ growth of N-doped carbon nanotubes from the products of graphitic carbon nitride etching by nickel nanoparticles
Source: Nanoscale Adv. 2024 Feb 21;6(6):1720–6. doi: 10.1039/d3na00983a (PMC10929602; doi:10.1039/d3na00983a)
Supplement: NA-006-D3NA00983A-s001 [file NA-006-D3NA00983A-s001.pdf]

Electronic Supplementary Material (ESI) for Nanoscale Advances.
This journal is © The Royal Society of Chemistry 2024

Supporting Information

In-situ Growth of N-Doped Carbon Nanotubes from the Products of Graphitic Carbon Nitride Etching by Nickel Nanoparticles

Mariusz Pietrowski,*[a] Emilia Alwin, [a] Michał Zieliński, [a] Sabine Szunerits, [b] Agata Suchora[a] and Robert Wojcieszak*[c]

a. Adam Mickiewicz University, Poznań, Faculty of Chemistry, Uniwersytetu Poznańskiego 8, 61-614 Poznań, Poland.

b. Univ. Lille, CNRS, Centrale Lille Univ. Polytechnique Hauts-de-France, UMR 8520 - IEMN, F-59000 Lille, France

c. Univ. Lille, CNRS, Centrale Lille, Univ. Artois UMR 8181 - UCCS - Unité de Catalyse et Chimie du Solide, F-59000 Lille, Fr

Corresponding Author

*Robert Wojcieszak, e-mail: Robert.wojcieszak@univ-lille.fr

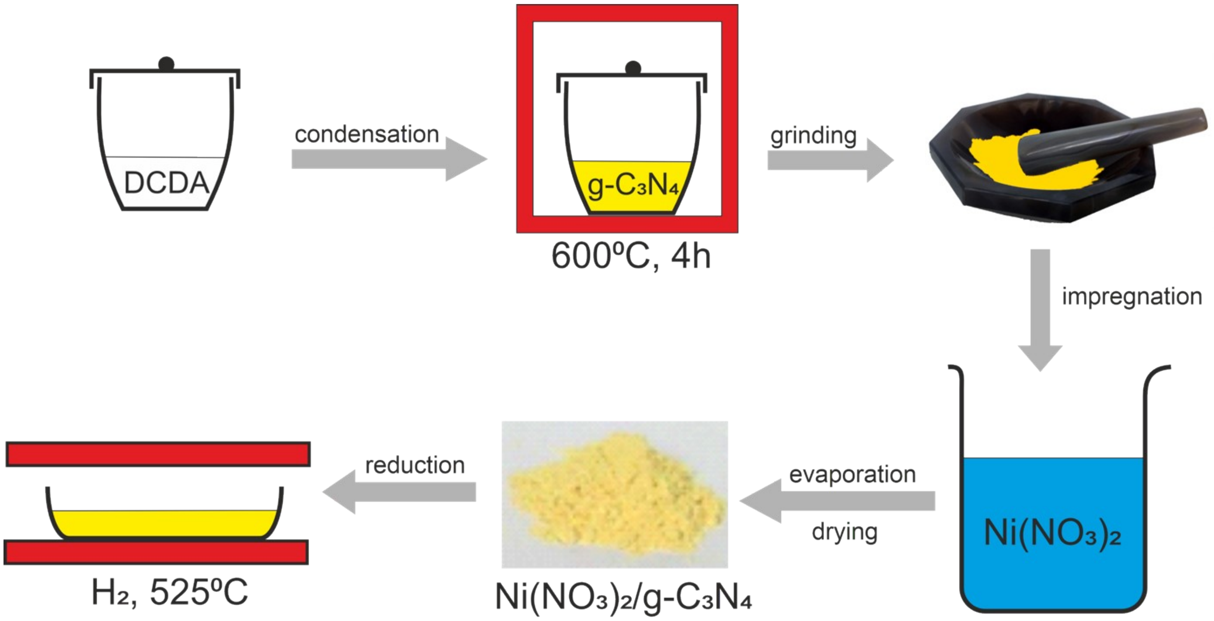

Figure S1. Scheme of catalysts preparation.

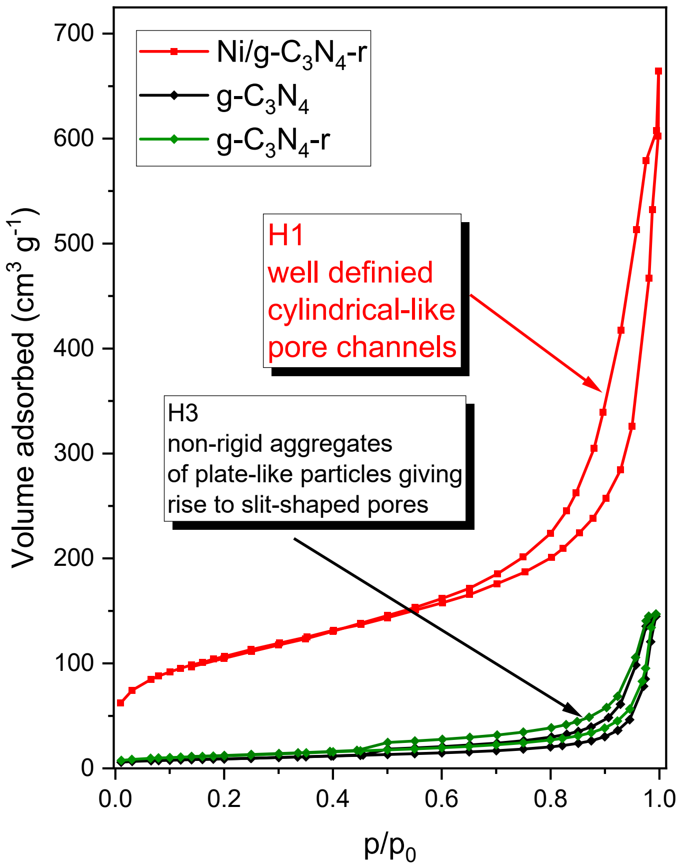

Figure S2. Low-temperature nitrogen adsorption/desorption isotherms of Ni/g-C_3N_4-r catalyst and pristine g-C_3N_4 and reduced g-C_3N_4.

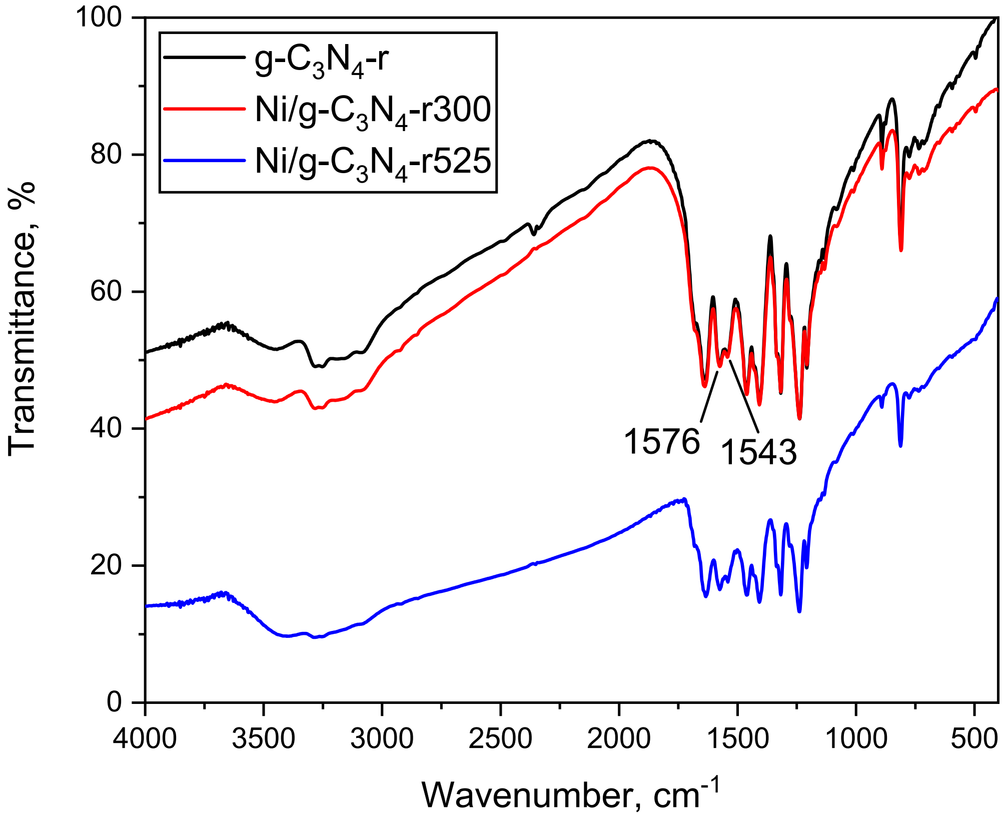

Figure S3. FTIR spectra of reduced graphitic carbon nitride (g-C_3N_4-r), and Ni/g-C_3N_4 sample reduced at 300 and 525 ^oC.

FTIR spectra of g-C_3N_4-r indicate a structure typical of graphitic carbon nitride. Bands in the 1100-1700 cm-1 range corresponding to characteristic stretching modes of the C-N ring of heterocycles were observed. The number and shape of the bands suggest the presence of tri-s-triazine (heptazine) subunits, as also indicated by the doublet at 1576 cm-1 and 1543 cm-1 [1]. A significant reduction in the intensity of FTIR bands in the 1100-1700 cm-1 range indicates partial degradation of the g-C3N4 structure after reduction of the Ni/g-C3N4 sample.

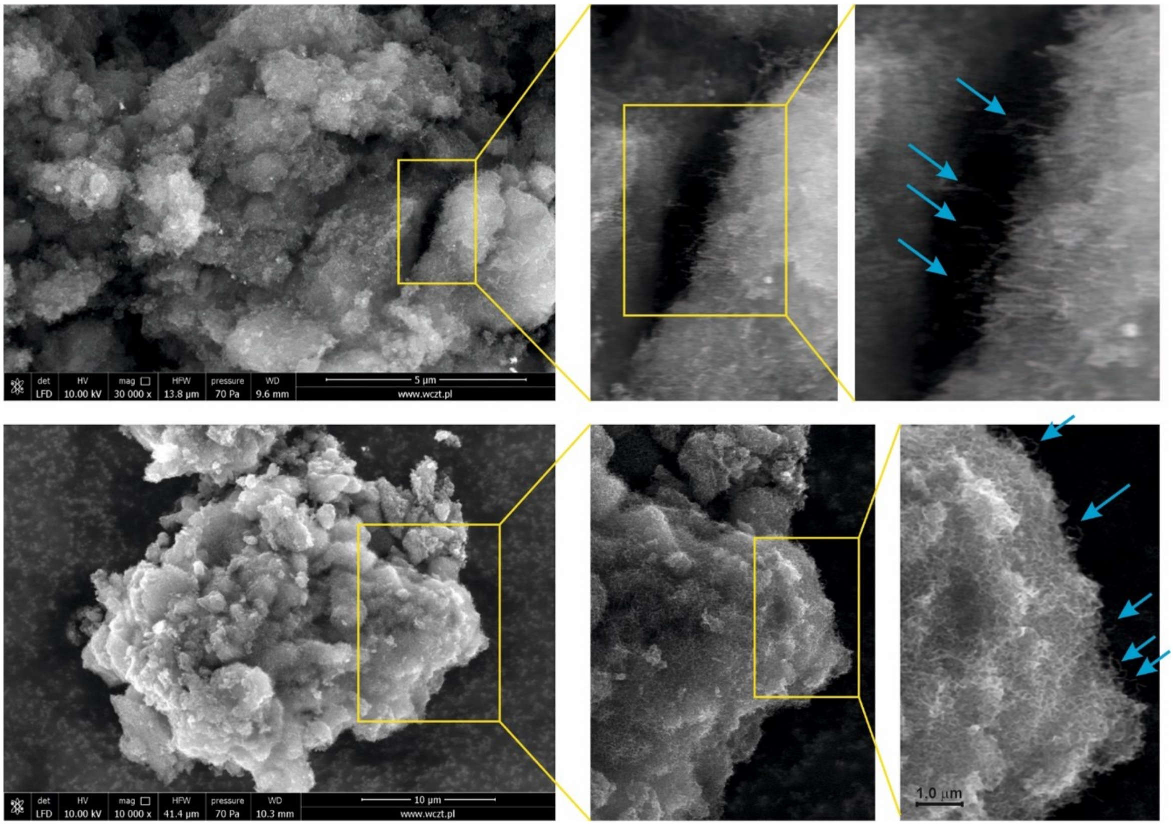

Figure S4. SEM images of Ni/g-C_3N_4-r sample (after reduction in hydrogen at 525 °C) and digital magnifications of selected areas. The fibrous structures are marked with blue arrows.

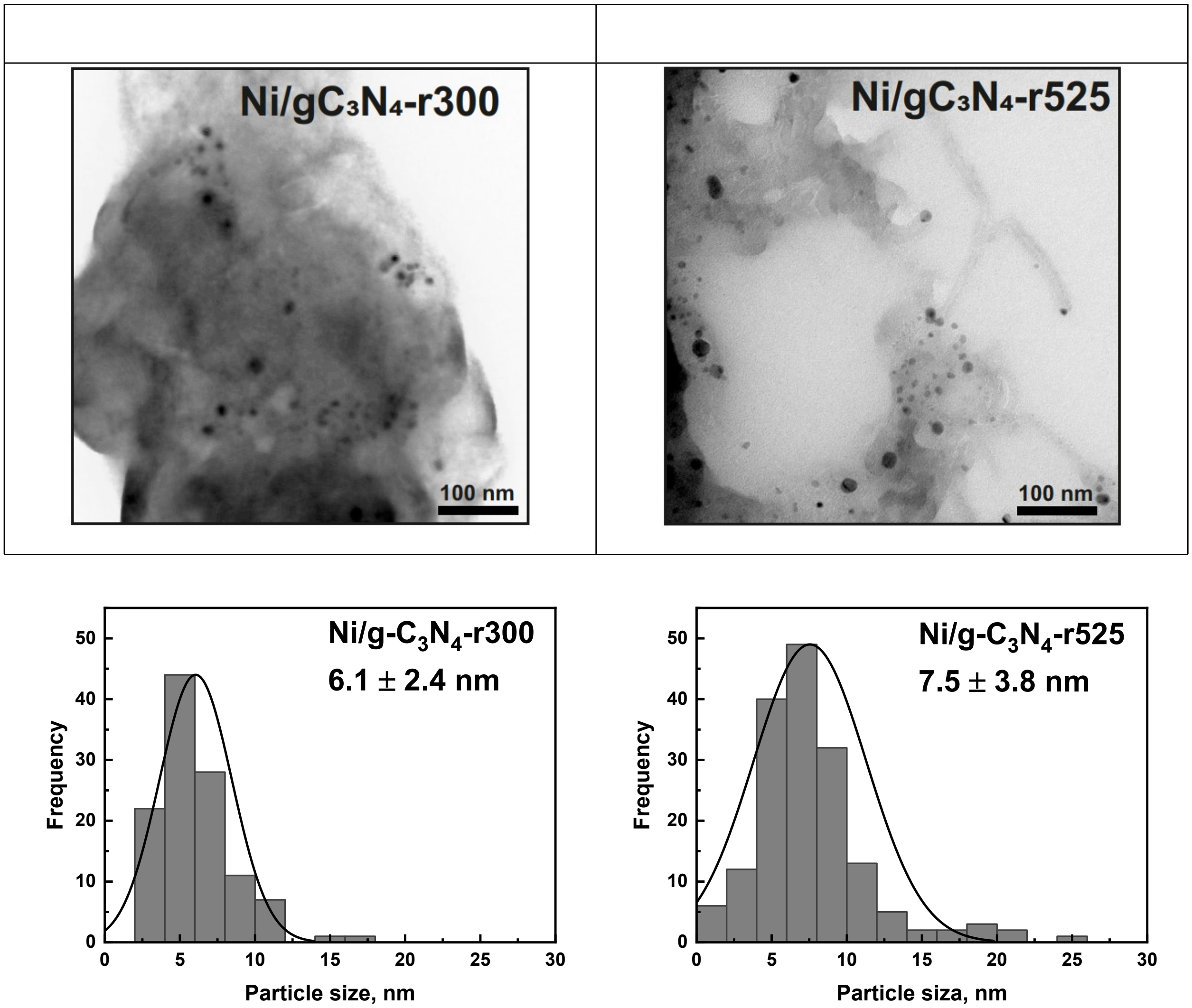

Figure S5. TEM images and particle size histograms of Ni/g-C_3N_4-r sample after reduction in hydrogen at 300 and 525 °C.

Identification of gases emitted upon the etching of Ni/g-C_3N_4

The gases released upon the reduction of Ni/g-C_3N_4 sample were identified with the use of a mass spectrometer ThermoStar made by Pfeiffer, model GDS301T2, with electron ionization and a QMA200 M analyser. The reduction of the Ni/g-C_3N_4 was performed in a ChemiSorb 2705 made by Micromeritics, in the flow of 10 vol.% H_2/Ar (30mL h^{-1}) at the temperature ramp of 10 ˚C min^{-1}. The spectrum presented in Figure S1 was taken upon the reduction at 525 ˚C. The fragmentation ions were identified on the basis of the NIST basis (National Institute of Standards and Technology) [2] and [3] – Figure S6, Table S1.

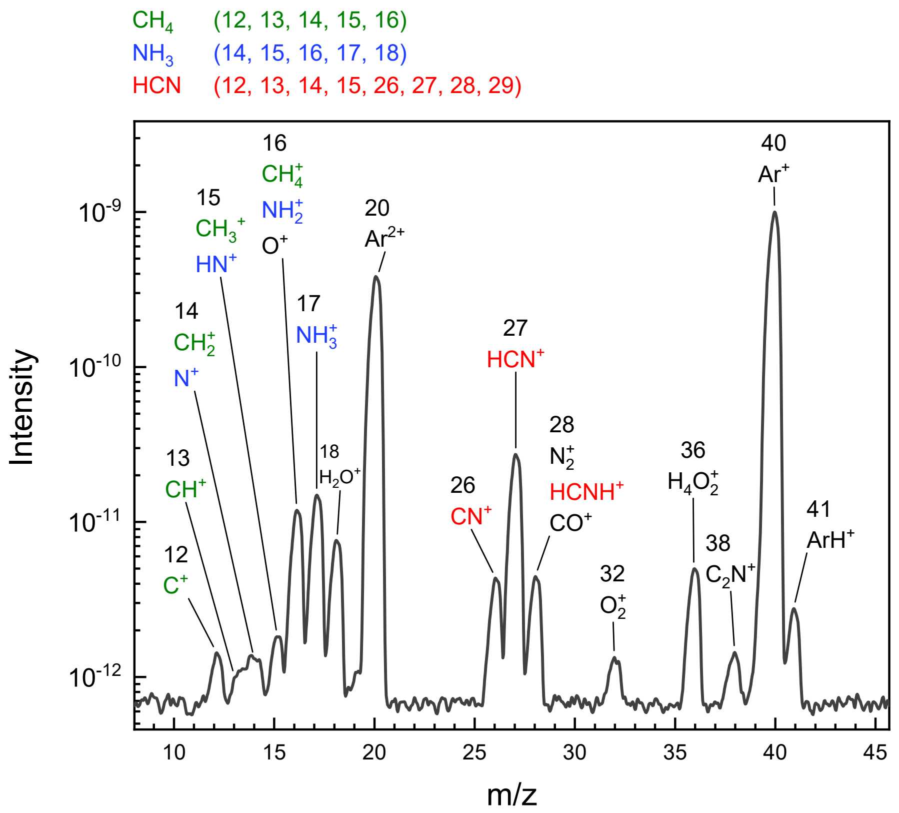

Figure S6. Mass spectrum recorded at 525 °C during temperature-programmed reduction of Ni/g-C_3N_4.

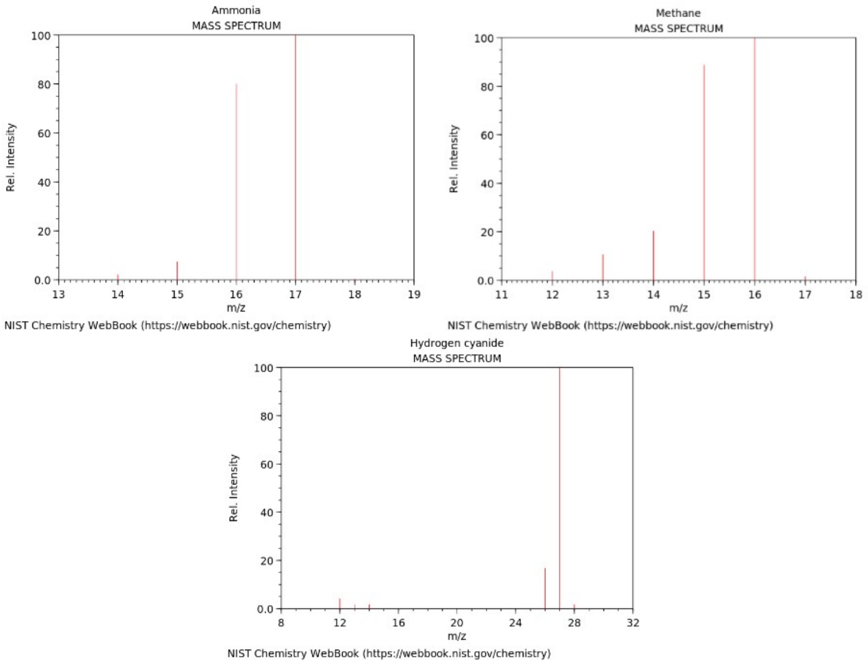

Figure S7. Reference mass spectra of ammonia, methane and hydrogen cyanide (according to [2]).

Table S1 presents the characteristic fragmentation ions of the gases observed in the mass spectra, and their relative intensities. In the gas products of the Ni/g-C_3N_4 sample reduction, the following ions were identified, those of m/z= 12, 13, 14, 15, 16 assigned to the fragmentation of methane, [3] those of m/z=14, 15, 16, 17, 18 assigned to ammonia [4] and those of m/z= 12, 13, 14, 15, 26, 27, 28, 29 evidencing the presence of hydrogen cyanide. Moreover, the spectra showed the ions coming from hydrogen and argon (components of the reducing mixture), small amounts of water and traces of air from the surroundings.

Table S1. NIST neutral gas fragmentation patterns for gases. G.T. Paschmann, P.W. Daly, I. International Space Science, Analysis methods for multi-spacecraft data, (1998) (http://www.issibern.ch/PDF-Files/analysis_methods_1_1a.pdf)

m/z	CH_4	NH_3	H_2O	HCN	N_2	O_2
12	0.038			0.042		
13	0.106			0.017		
14	0.204	0.022		0.017	0.137	
15	0.888	0.075		0.001		
16	1.000	0.801	0.009			0.218
17	0.016	1.000	0.212			
18		0.004	1.000			
19			0.005			
20			0.003			
26				0.168		
27				1.000		
28				0.017	1.000	
29				0.001	0.007	
32						1.000

Table S2. The results of the XPS analysis of g-C_3N_4, g-C_3N_4-r and Ni/g-C_3N_4-r after reduction at 525 °C.

		g-C_3N_4			g-C_3N_4-r			Ni/g-C_3N_4		
		B.E., eV	FWHM	% at.	B.E., eV	FWHM	% at.	B.E., eV	FWHM	% at.
Nitrogen	Quaternary	401.2	1.0	4.2	401.2	1.0	4.2	401.2	1.7	1.0
	NH	400.3	1.0	5.0	400.3	1.0	5.5	400.4	1.7	0.4
	NH_2	399.3	1.0	6.5	399.4	1.0	6.9	399.4	1.7	0.8
	Py	398.6	1.0	27.9	398.6	1.0	26.0	398.7	1.7	2.5
	$N(O_x)$	-	-	-	-	-	-	404.1	2.2	0.4
	Total nitrogen:			43.6			42.6			5.1
Carbon	O-C=N	-	-	-	-	-	-	289.2	0.6	0.8
	Py	288.0	1.0	34.9	288.0	0.9	35.1	288.1	1.1	1.8
	Nitrile (C≡N)	287.0	0.3	0.1	287.1	1.1	1.1	-	-	-
	AdC	284.6	1.1	17.3	284.6	1.4	18.6	284.6	1.1	21.6
	Graphitic	-	-	-	-	-	-	284.4	0.9	53.9
	Defective	-	-	-	-	-	-	283.9	1.4	10.8
	Total carbon*:			35.0			36.2			67.3
Oxygen	O_2 ads.	533.1	1.4	2.1	533.7	1.6	1.1	533.9	1.4	1.5
	H_2O ads.	531.7	1.2	1.9	532.2	1.6	1.6	532.5	1.4	1.9
	NiO	530.2	1.3	0.1	-	-	-	529.7	0.9	0.2
	O=C–N	-	-	-	-	-	-	531.5	0.9	0.1
	NiO	-	-	-	-	-	-	531.3	1.7	0.9
	Total oxygen:			4.1			2.7			4.6

*without of AdC

References

(1) Lotsch, B.V.; Schnick, W. From triazines to heptazines: Novel nonmetal tricyanomelaminates as precursors for graphitic carbon nitride materials. Chem. Mater. 2006, 18, 1891–1900. DOI: 10.1021/cm052342f

(2) NIST Chemistry WebBook, NIST Standard Reference Database Number 69. National Institute of Standards and Technology. August 25, 2021. https://doi.org/10.18434/T4D303 (accessed.

(3) Wei, B.; Zhang, Y.; Wang, X.; Lu, D.; Lu, G. C.; Zhang, B. H.; Tang, Y. J.; Hutton, R.; Zou, Y. Fragmentation mechanisms for methane induced by 55 eV, 75 eV, and 100 eV electron impact. *J. Chem. Phys.* **2014**, *140* (12), 8, Article. DOI: 10.1063/1.4868651.

(4) Rejoub, R.; Lindsay, B. G.; Stebbings, R. F. Electron-impact ionization of NH_3 and ND_3. *J. Chem. Phys.* **2001**, *115* (11), 5053-5058. DOI: 10.1063/1.1394748. Wuest, M.; Evans, D. S.; von Steiger, R. Fragmentation Patterns and Total Ionization Cross Sections. In *Calibration of Particle Instruments in Space Physics*, Wuest, M., Evans, D. S., von Steiger, R. Eds.; ESA Publications Division Keplerlaan 1, 2200 AG Noordwijk, The Netherlands, 2007; pp 555-570.
